# Two-stage treatment of acetabular bone defect in tuberculosis of the hip by intended ankylosis followed by total hip arthroplasty: a case report

**DOI:** 10.1186/1757-1626-2-6532

**Published:** 2009-03-25

**Authors:** Els E Vogelpoel, Jurjen J Been, Arthur A de Gast

**Affiliations:** 1 Department of Orthopedic Surgery, Vrije Universiteit Medical Center, de Boelelaan, 1117, Room 3F043, 1081 HV Amsterdam, Netherlands

## Abstract

### Introduction

In case of severe post-tuberculosis osteoarthritis of the hip, arthrodesis, excision arthroplasty, or total hip arthroplasty may be considered. The latter can be challenging, because destruction of the joint, and most importantly the acetabulum, is frequently seen. To fill up acetabular bone stock loss during total hip arthroplasty, there is the possibility to use bone auto-grafts and allografts. Complications are graft rejection, mechanical failure of implants and gradual migration of the cup into the graft. Other options for creating a stable acetabular component in total hip replacement are screw fixation of the acetabular component or using a stemmed acetabular component. An alternative is the use of an anti-protrusion cage, for which the risk of loosening however is known. In young patients especially, such solution are not always appealing. Therefore, we created an intended ankylosis of the hip joint to fill up the acetabular bone loss with the patients own femoral head. To our knowledge this treatment strategy has not been described before.

### Case presentation

We present a 33-year-old Caucasian woman with an acetabular bone defect caused by tuberculous arthritis of the left hip joint. Instead of performing a resection arthroplasty followed by total hip arthroplasty in a second stage, we decided to intentionally ankylose the hip joint in order to fill up the acetabular defect with the patientâ€™s own femoral head. Two years after the start of a one year course of tuberculostatic chemotherapy, we took down the ankylosed hip and placed an uncemented total hip prosthesis. The technical and functional outcome of this procedure appeared to be very favourable, the acetabular defect was filled up and the bone remodeled completely.

### Conclusion

In order to resolve the problem of acetabular osseous defects in tuberculous arthritis of the hip, intended spontaneous fusion of the femoral head with the acetabular can be a favorable treatment strategy. Subsequently this situation was used as a solid base for the acetabular component of the total hip prosthesis. It resulted in a optimal acetabular bone stock during acetabular component implantation with a very good technical and clinical outcome at 40-months follow up It is understood that this method may not be applicable to all resembling patients. However, this solution may be considered worthwhile in individual cases.

## Introduction

Over the past 20 years, an increasing number of tuberculosis infections has been reported worldwide [1]. In approximately 20% of all cases, this concerns extrapulmonary tuberculosis, including skeletal localisations (2%) [2]. Tuberculosis of the hip joint constitutes approximately 15% of all cases of osteoarticular tuberculosis.

Skeletal tuberculosis most frequently occurs during the first three decades of life. The characteristics are insidious onset, mono-articular or single-bone involvement, and systemic symptoms. The diagnosis can be made on clinical and radiologic examinations and semi-invasive investigations.

The cornerstone of treatment is multidrug antituberculous chemotherapy for 12 to 18 months combined with exercises of the involved joint throughout the period of healing. In selected cases, surgery may be required. When surgery becomes the therapeutic modality of choice, antituberculous chemotherapy remains necessary in the prevention of reactivation of the tuberculosis [3].

In case of severe post-tuberculosis osteoarthritis of the hip, arthrodesis, excision arthroplasty, or total hip arthroplasty may be considered. The latter can be challenging, because destruction of the joint, and most importantly the acetabulum, is frequently seen.

To fill up acetabular bone stock loss, there is the possibility to use bone auto-grafts and allografts. Complications are graft rejection, mechanical failure of implants and gradual migration of the cup into the graft. Other options for creating a stable acetabular component in total hip replacement are screw fixation of the acetabular component or using a stemmed acetabular component. An alternative is the use of an anti-protrusion cage, for which the risk of loosening however is known. In this report we present a 33-year old patient who had tuberculosis arthritis of the hip with extensive acetabular osseous defects. Because all methods mentioned above have disadvantages to some degree [4]-[6] we decided to create a usable acetabular bone stock by intentionally ankylosing the hip and thereby filling up the bone loss with the patients own femoral head.

## Case presentation

In October 2001, a 33-year-old Caucasian female office employee visited our orthopedic outpatient clinic, with complaints of chronic pain in the left hip since 1997. Her medical history showed two episodes of pleuritis and infertility problems. The patient lived a healthy life; non-smoker and 2 units of alcohol per week. Her length was 1.68 m and her weight approximately 67 kg. In both episodes of pneumonia Ziehl-Nielsen staining showed no tubercles in pleural effusion.

In order to find an explanation for her infertility the patient had undergone a laparoscopy in 1999. Intra-abdominal granulomas, adhesions and signs of chronic peritonitis were found. Ziehl-Nielsen and periodic acid-schiff staining (PAS staining) of peritoneal effusion and granulomas did not show acid fast bacilli.

One year previously a clinical analysis of her hip complaints was performed on the rheumatology department of another hospital. There, the hip had been visualized by CT scan, MRI and skeletal scintigraphy, but no diagnosis had come up. Rheumatologic blood testing and Mycoplasma serology had been negative. Ziehl-Nielsen staining of pus aspired from the hip had been negative for acid fast bacilli, culture had been negative for *Mycobacterium tuberculosis* as had polymerase chain reaction.

**Figure 1:**
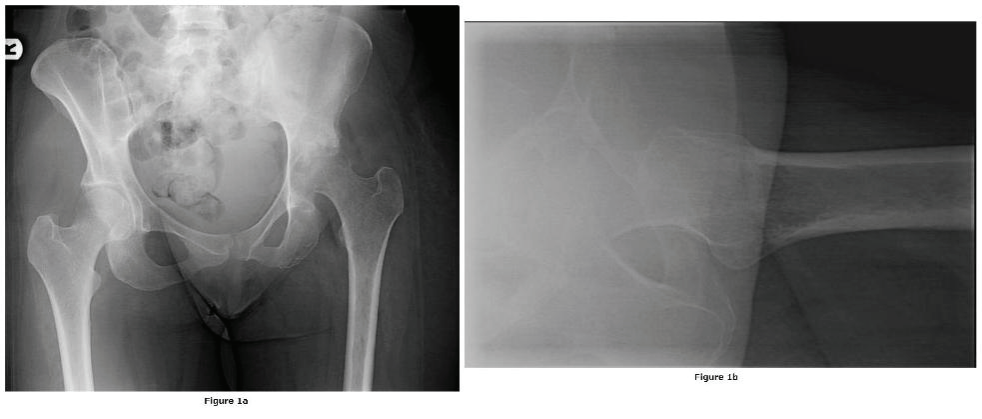
**(a) Preoperative radiograph of the pelvis showing erosive destruction of the left hip joint with a superior migration of the femoral head indicating a significant acetabular bone loss. (b)** Preoperative lateral radiograph of the left hip showing irregularities on the site of the femoral head and the same superior migration

**Figure 2:**
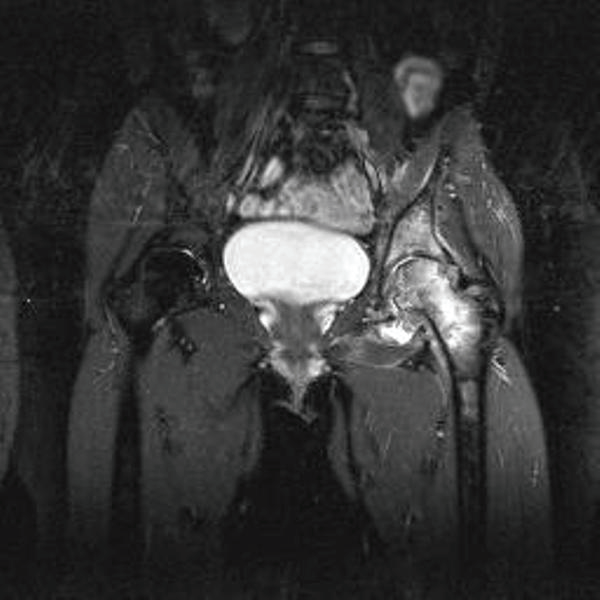
**MRI scan made 2 years earlier already shows oedema of the bone marrow, joint effusion and some erosive changes of the left hip joint**.(STIR-image; TR 5700.0 ms, TE 30.0 ms)

When the patient first visited us, her walking distance was limited to thirty minutes with crutches. No other joints were affected. No fever, nocturnal sweating or weight loss was present. On physical examination, there were no symptoms of infection. All hip joint movements were limited and painful (flexion 80Â°; abduction 20Â°; adduction 10Â°; internal rotation 0Â°; external rotation 0Â°). Hematological blood testing revealed a total white blood count of 9.4/cu mm. and ESR of 30 mm in the first hour. Conventional hip radiography showed some osseous destruction of the joint with narrowing of the joint space, suggesting loss of articular cartilage (Figure 1a and Figure 1b). A review of the earlier made MRI revealed osteonecrosis, destruction of the hip joint, periarticular oedema, and multiple fluid collections, and with these features and the abdominal granulomas in mind the suspicion of articular tuberculosis arose (Figure 2). Additional chest radiography did not show any abnormalities. We decided to perform an open biopsy to obtain a diagnosis. At surgery, granulation tissue and destruction of the cartilage of the femoral head was seen, also suggesting articular tuberculosis. In comparison with the earlier made MRI, which showed some acetabular destruction, there was progressive destruction of the superior part of the acetabulum which had resulted in a large local osseous defect and superior migration and lateralization of the femur. Our goal was to fill the acetabular osseous defect by in situ ankylosis of the femoral head, instead of performing the classical Girdlestone resection arthroplasty. After soft tissue debridement, the left hip was immobilized in a hip-spica cast.

**Figure 3:**
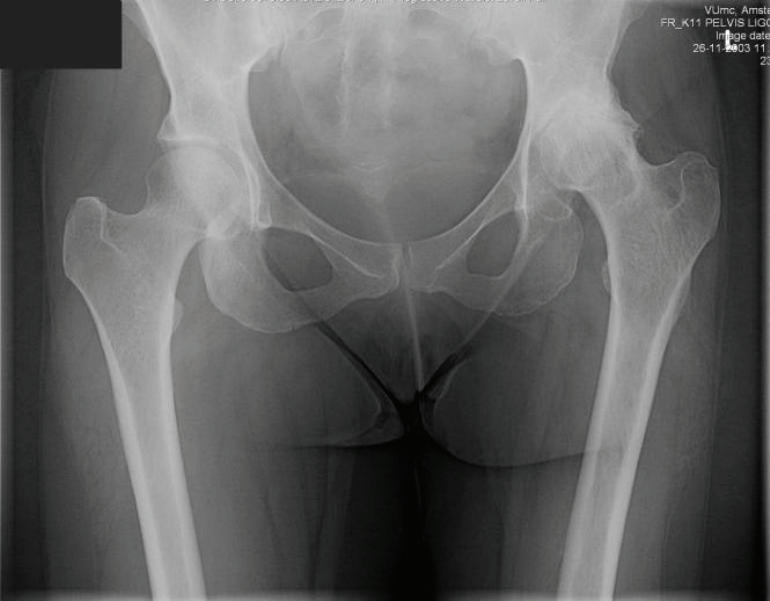
**Radiograph of the pelvis shows ankylosis of the left hip joint**. The femoral head filled up the acetabular defect.

Ziehl-Nielsen staining of the debris was positive for acid-fast bacilli. A Mantoux-test was performed, which was strongly positive. The patient was treated with tuberculostatics (Isoniazid, Rifampicine, Ethambutol and Pyrazinamide) for 12 months. Filling of the acetabular defect resulting from ankylosis with the femoral head occurred approximately 4 months after initiation of chemotherapy and immobilisation (Figure 3). After fusion, shortening of the left leg and an intentional flexion position of 20Â° were present. From then the patient was mobilized without crutches. In November 2003, two years after the index operation, a primary one-stage cementless total hip arthroplasty was performed (OsteonicsÂ© Total Hip System, Stryker USA). Histopathological examination of the retrieved bone and joint capsule showed no signs of tuberculosis. Therefore, postoperatively she did not receive any tuberculostatics. No peri or postoperative complications occurred.

**Figure 4:**
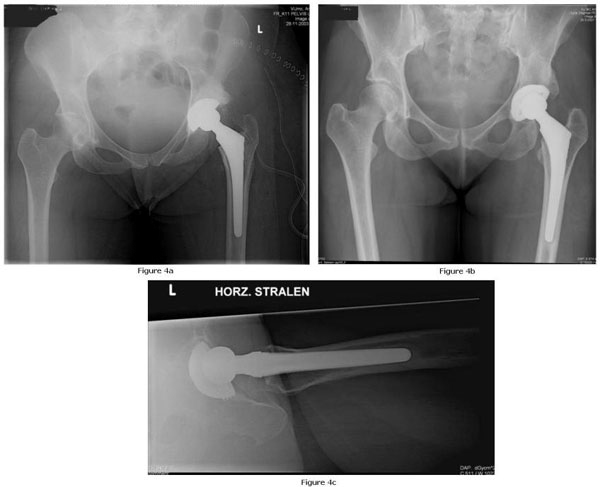
**(a) Postoperative radiographs of the pelvis at 6-months follow up shows good position of hip prosthesis**. **(b)** Postoperative radiograph of the pelvis at 40 months follow up shows further remodeling of acetabular bone stock. **(c)** Postoperative radiographs lateral film at 40 months follow up shows good position of the hip prothesis

At the latest follow up in March 2008, 52 months after total hip replacement, no signs of reactivation of the tuberculosis were present. The patient experienced no pain and had a normal range of motion. She did not suffer from any significant limitations in her daily activities, including sports and labour. The bone that formerly belonged to the femoral head, had fully integrated with the acetabulum. Radiological assessment of the left hip showed no signs of loosening (Figure 4a-c).

## Discussion

Literature does not provide consensus on the preferred treatment of advanced tuberculous coxarthritis in young patients [7]. Hip arthrodesis is a viable treatment technique to relieve pain and thus obtain functional improvement. With current internal fixation techniques, a fusion rate of over 80% can be achieved with maximal preservation of bone stock.

Proper patient selection and optimal arthrodesis position are essential for successful long-term results [8]. However, a long-term hip arthrodesis can cause lower back pain and ipsilateral knee pain. Many patients will eventually require a takedown of the fused hip and conversion to a total hip arthroplasty [9]. Patients generally can expect an improvement in function and mobility from converting a fused hip to a total hip arthroplasty. However, the technically demanding nature of the procedure should not be underestimated. Patients should be cautioned with regard to the possibility of a higher rate of complications than seen with primary total hip arthroplasty [10]**.** As reported by Hamadouche et al. in 2001, the functional outcome of hip arthroplasty after hip fusion takedown is related to the intraoperative status of the hip abductor muscles [11], in our patient however we didnâ€™t expect this to be a problem because of the relative short time of the intended ankylosis and the excellent state of the hip abductor muscles.

As reported in the literature of the past 15 years, arthroplasty has become a more and more accepted treatment for patients with tuberculosis of the hip. Some reluctance to include hip arthroplasty in their treatment of tuberculous coxitis is still common among orthopedic surgeons, because of the possibility of reactivation of the disease. In spite of the lack of evidence based upon randomized clinical trials concerning the effectiveness of tuberculostatics in reducing the risk of reactivation, we found consensus in literature that administration of tuberculostatics is preferable.

Kim et al. in 2001 reported about 60 cases of long-standing hip tuberculosis treated with total hip arthroplasty. Some patients did not receive tuberculostatic chemotherapy. The longest follow up was more than 27 years. Prosthetic life was limited to at most 20 years in their longest cases mainly because of loss of fixation of the acetabular component. There were recurrences of tuberculosis in 5 hips, but these cases were not patients without tuberculostatic chemotherapy. Still, Kim et al. confirmed that antituberculous chemotherapy is crucial in total hip arthroplasty reconstruction for tuberculous coxartritis [12]. Eskola (1988) reported the results of cementless total joint replacement in 18 patients with long-standing (on average 34 years after the onset of infection) tuberculosis of the hip. Mean follow-up was 3.5 years. Seven of the patients received anti-tuberculous drugs. None of the patients developed reactivation of the disease. Despite the absence of any reactivation of tuberculosis in this series, they recommend the use of specific prophylaxis [13].

Acetabular bone deficiency caused by tuberculosis can present a challenge during total hip arthroplasty, especially in young patients. When major segmental defects are present and prosthetic stability is not possible in host bone, structural auto or allografts may be necessary to satisfy the principles of acetabular reconstruction. Using quality bone, proper fixation, and buttressing of structural grafts against host bone, a high degree of success can be expected [14].

Schreurs et al. studied if this bone impaction grafting technique could provide long-term prosthesis survival in deformed and irregular acetabula. 51 acetabular reconstructions were performed in 48 relatively young patients. Follow up was 3 to 18 years. Schreurs found low complication and reoperation rates. Schreurs et al. concluded that acetabular reconstruction with the use of impaction bone-grafting and cemented cup is a reliable and durable technique, associated with good long-term results in young patients with acetabular bone stock defects [15,16].

An alternative procedure involves preparation of the acetabulum, filling the defect with bone auto-grafts, placement of a Burch-Schneider cage, fixation with screws on the lateral wall and placement of a cement and plastic cup. Satisfactory results of this procedure were observed by Simeonydes et al., indicating that effective support of the acetabulum can be achieved using Burch-Schneider cages [17]. Their patients did not suffer from tuberculosis however in our patient acetabular bone destruction complicated the possibility of a one-stage total hip arthroplasty. In order to resolve the problem of acetabular osseous defects, we aimed for spontaneous fusion of the femoral head with the acetabular remains while admitting chemotherapy. Cast immobilization of the affected leg attributed to an ankylosed hip in a favorable position and the osseous acetabular defect was filled by the femoral head bone mass. Subsequently this was used as a solid base for the acetabular component of the total hip prosthesis.

Although grave acetabular destruction may give rise to considerable doubt in relationship to the biomechanical outcome of total hip arthroplasty, our patient still is free of complications and functioning well in daily life and work after a follow up of 52 months. It is understood that this method may not be applicable to all resembling patients. However, this solution may be considered worthwhile in individual cases.

## List of abbreviations

MRI, Magnetic Resonance Imaging; CT, Computer tomography; ESR, Erythrocyte sedimentation Rate; PAS, Periodic Acid Schiff.

## Consent

Written informed consent was obtained from the patient for publication of this case report and accompanying images. A copy of the written consent is available for review by the Editor-in-Chief of this journal.

## Competing interests

The authors declare that they have no competing interests.

## Authorsâ€™ contributions

EV and JB performed acquisition, analysis and interpretation of data and drafted the manuscript. ADG performed the surgeries, coordinated the study, performed interpretation of data and took part in preparation of the manuscript.
